# How Does Industrial Waste Gas Emission Affect Health Care Expenditure in Different Regions of China: An Application of Bayesian Quantile Regression

**DOI:** 10.3390/ijerph16152748

**Published:** 2019-08-01

**Authors:** Xiaocang Xu, Zhiming Xu, Linhong Chen, Chang Li

**Affiliations:** 1 Upper Yangtze river economic research center/School of Economics, Chongqing Technology and Business University, Chongqing 400067, China; 2 Department of Business, ESCP Europe Business School, 75011 Paris, France; 3 School of Mathematics and Statistics, Chongqing Technology and Business University, Chongqing 400067, China; 4 School of Public Administration, Sichuan University, Chengdu 610065, China; 5 College of Business Administration, Wonkwang University, Jeonbuk 54538, Korea

**Keywords:** Bayesian quantile regression (BQR), health care expenditure (HCE), industrial waste gas emission (IWGE), double heterogeneity, regional difference

## Abstract

Industrial development has brought about not only rapid economic growth, but also serious environmental pollution in China, which has led to serious health problems and heavy economic burdens on healthcare. Therefore, the relationship between the industrial air pollution and health care expenditure (HCE) has attracted the attention of researchers, most of which used the traditional empirical methods, such as ordinary least squares (OLS), logistic and so on. By collecting the panel data of 30 provinces of China during 2005–2016, this paper attempts to use the Bayesian quantile regression (BQR) to reveal the impact of industrial air pollution represented by industrial waste gas emission (IWGE) on HCE in high-, middle-, low-income regions. It was found that double heterogeneity in the influence of IWGE on HCE was obvious, which revealed that people in high-, middle-, low-income regions have significantly different understandings of environmental pollution and health problems. In addition, the BQR method provided more information than the traditional empirical methods, which verified that the BQR method, as a new empirical method for previous studies, was applicable in this topic and expanded the discussion space of this research field.

## 1. Introduction

It is widely recognized that China has achieved great improvements in economic growth and population health in the last few decades, such as a dramatically lower mortality rate, increased life expectancy and extensive immunization coverage. The latest statistics show that the national infant mortality rate was 7.5 per thousand in 2016, which was a decrease by 76.7 percent from 2000. At the same time, the rate of nationwide hospital births reached 99.7 percent in 2016, which was an increase by 36.9 percent from 2000. However, increasing environmental problems have led to serious health problems. With the promotion of industrial policy and the development of the economy and technology, economic development has entered the middle and late stages of industrialization in China. However, the current environmental problems are still not optimistic, and there is still a considerable way to go to control pollution and accelerate to meet the peak of the environmental Kuznets curve (EKC) although new energy development has attracted worldwide attention [1,2]. Industrial pollution to the environment is more serious compared to others such as agriculture, urban construction and people’s lives [3,4,5,6]. Environmental pollution, especially industrial waste gas emission (IWGE), results in disorders of the human immune system and therefore causes health risks, notably high occurrences of chronic diseases such as hypertension, heart disease, chronic pulmonary disease and diabetes [7,8]. In China, IWGE reached 685,190 billion cubic meters in 2016, and had 268,988 billion cubic meters in 2005. Meanwhile, the number people with chronic diseases increased from 318.73 million in 2005 to 337.05 million in 2016.

Several studies from medical science provide evidence that waste gas emissions affect some types of chronic diseases and disabilities. For instance, sulfur dioxide and particulates respectively lead to a 3–4% and a 2% increase in mortality in Koln [9]. PM2.5 emissions were significantly associated with the incidence of ischemic heart disease and mortality by using PM2.5 data from 2010 to 2012 in Beijing [10]. There have also been several scholarly attempts to find scientific evidence on the relationship between waste gas emissions and health care. For example, exposure to high environmental levels of PM2.5 might lead to the disproportional risk of type 2 diabetes in the mainland USA [11]. Nayak and Chowdhury [12] proved that there was a positive and significant relationship between waste gas emissions level and the days of respiratory illnesses in Odisha of India, which showed that the significant impact of environmental pollution on health problems has been determined. A few relevant studies were also undertaken in China. For example, Li et al. [13] measured the economic loss related to health problems due to waste gas emissions and found that the economic loss brought by PM10 and SO_2_ accounted for 1.63% and 2.32% of the GDP in 74 cities respectively. Liu et al. [14] evaluated the health care burden of waste gas emissions and heavy metals in the spring and winter of 2016.

Thus, the linkage between environmental emissions and health care expenditure (HCE) has aroused great interest among scholars around the world, especially in developed countries. Ridker [15] firstly evaluated the economic loss of environment pollution on HCE. For decades, a number of researchers found the positive relationship between environmental pollution and HCE [16,17,18,19,20]. Note that, some relative studies were included in the tripartite relationship of economic growth, environmental emissions and HCE. For instance, the dynamic links between CO_2_ emissions, health spending and GDP growth were used in the data of 51 countries [21,22,23,24]. On the other hand, the relevant research literature has also emerged in China in recent years [25,26,27].

Some different empirical methods have been used in this field. For example, Jerrett et al. [28] adopted the sequential two-stage regression model to examine the relationship between pollution trends and health expenses in 49 counties in Ontario. Narayan and Narayan [18] used a panel co-integration approach and found that CO emissions had a significant positive influence and income had an elastic influence on HCE in the long term. Chaabouni [29] used dynamic simultaneous equation models to compare the relationship between CO_2_, HCE and economic growth in lower and higher countries during 1995–2013. Nicholas et al. [30] used the panel quantile regression methodology to analyze U.S. state-level CO_2_ emissions and its effect on HCE. Chinese scholars also adopted a quantile regression approach to a panel data analysis of health-care expenditure in organizations for economic cooperation and development countries [31].

Therefore, the academic research on the environment pollution and HCE has been increasing quickly and the relative empirical methods have developed more maturely, such as the panel data model and the OLS regression. However, little literature has been attempted to use quantile regression method [30]. In particular, there was rarely any relative literature which adopted the quantile regression with the Bayesian approach so far, which has some advantages over OLS or the least squares dummy variable model (LSDV). For example, it does not require the assumption of the independent and identically distributed error terms which the OLS assumptions do not hold [32]. There is still some room for further discussion and expansion with BQR method used in this field, although it has been investigated intensively and theoretically in many other fields, such as biomedical applications. In view of this, this paper aims to offer a new empirical research method of a Bayesian quantile regression (BQR) to evaluate the relationship between environmental pollution and HCE in different income regions in China. This paper applied the BQR method for the influence of IWGE on HCE, and revealed some important findings. 

The paper is organized as follows: The BQR method and the data source are detailed in Section 2. Next, the empirical results of BQR are provided in section Results. Finally, the discussion and the conclusion of the empirical analysis are examined in section Discussion and section conclusions. 

## 2. Materials and Methods 

### 2.1. Estimation Method: BQR

Quantile regression has become an important and popular empirical research tool to study the conditional response distribution in regression [33]. The general path for deriving quantile regression is based on the standard linear model and quantile regression is achieved by extending the median case to all other arbitrary quantiles. The method of R package-quantreg has been widely used to minimize the objective function [34]. However, it has computational requirements in large statistical applications. The R package-quantreg presents an interior point approach for larger problems. The confidence intervals are usually calculated by using a simplex algorithm to invert the rank score test.

Koenker and Machado [35] were the first to use independently the distributed asymmetric Laplace densities (ALD) to solve the minimization problem. Yu and Zhang [36,37] showed a three-parameter ALD with a skewness parameter for quantile modeling. The Bayesian implementation of the quantile regression begins by forming a likelihood comprised of independent ALD. Next, the quantile of interest has to be specified and priors should be put on the model parameters. In the relevant literature, the mixed OLS estimation or the fixed effect model has been used extensively to consider the determinants of health expenditures, and only a few studies have attempted to use the quantile regression method [30,38]. Quantile regression can offer more additional flexibility than OLS. For example, it does not require the assumption of independent and identically distributed error terms where the OLS assumptions do not hold. The Bayesian ALD approach does assume independent and identically distributed errors and contrasts with traditional quantile regression [39,40,41]. Recently, the Bayesian approach for quantile regression models are used with both adaptive lasso and without adaptive lasso [32].

The novelty of this paper is the attempt of a new empirical method in the existing research fields. It is well known that the Bayesian method is mainly used in micro-data set analysis, such as medical experiments. This paper attempts to apply it to macro data analysis, although there may be many immature problems that need to be solved in the future. It should be noted that the authors do not want to prove the BQR method is very superior to the traditional empirical methods, but only hope to introduce the BQR method into this research field as a new empirical method on the basis of previous studies to expand the discussion space of this field. Our empirical research mainly includes two parts. First, the model is set up as BQR to obtain its empirical results. Second, this study compared with the regression results of BQR, OLS, quantile regression (QR) and Bayesian linear regression (BLR). The detailed mathematical formula derivation of the BQR method can be provided by the R Package-Bayes QR for reference [32].

### 2.2. Model Construction, Variable Selection and Data Sources

By collecting the panel data of 30 provinces of China except for Tibet, Taiwan, Hong Kong and Macao during 2005–2016, this paper estimated the long-term impact of industrial air pollution represented by IWGE on HCE. To examine the heterogeneity of different regions, 30 provinces were divided into high-, middle-, low-income region based on the per capita income in 2016 and regression model was built (Formula 1–4) for BQR analysis. 

The regression models constructed were as follows:

(1) $$\begin{array}{cc} \ln(HCE_{Wt})= & \beta_{0}+\beta_{1}\ln(INCOME_{t})+\beta_{2}\ln(IWGE_{t})+\beta_{3}\ln(DCLI_{t})\\ & +\beta_{4}\ln(GFE_{t})+\beta_{5}\ln(ODR_{t})+\beta_{6}\ln(CD_{t})+\beta_{7}\ln(HT_{t})+\varepsilon_{i} \end{array}$$

(2) $$\begin{array}{cc} \ln(HCE_{Ht})= & \beta_{0}+\beta_{1}\ln(INCOME_{t})+\beta_{2}\ln(IWGE_{t})+\beta_{3}\ln(DCLI_{t})\\ & +\beta_{4}\ln(GFE_{t})+\beta_{5}\ln(ODR_{t})+\beta_{6}\ln(CD_{t})+\beta_{7}\ln(HT_{t})+\varepsilon_{i} \end{array}$$

(3) $$\begin{array}{cc} \ln(HCE_{Mt})= & \beta_{0}+\beta_{1}\ln(INCOME_{t})+\beta_{2}\ln(IWGE_{t})+\beta_{3}\ln(DCLI_{t})\\ & +\beta_{4}\ln(GFE_{t})+\beta_{5}\ln(ODR_{t})+\beta_{6}\ln(CD_{t})+\beta_{7}\ln(HT_{t})+\varepsilon_{i} \end{array}$$

(4) $$\begin{array}{cc} \ln(HCE_{Lt})= & \beta_{0}+\beta_{1}\ln(INCOME_{t})+\beta_{2}\ln(IWGE_{t})+\beta_{3}\ln(DCLI_{t})\\ & +\beta_{4}\ln(GFE_{t})+\beta_{5}\ln(ODR_{t})+\beta_{6}\ln(CD_{t})+\beta_{7}\ln(HT_{t})+\varepsilon_{i} \end{array}$$

Where *t* refers to the time period, and refers to 2005–2016. Formula 1–4 respectively represent the regression model for the whole country, the high-income region, the middle-income region and the low-income region.

By referring to a number of previous studies, such as Nicholas et al. [30] and Tian et al. [31], this paper selected HCE as the dependent variable, and IWGE, income, government financial expenditure and so on as the independent variables. It is worth noting that the density of commercial life insurance and chronic diseases were introduced to be independent variables since they had a positive impact on the HCE. More specifically, the dependent variable was per capita individual HCE, the independent variable was per capita IWGE, and the control variables included were per capita income, chronic diseases, and per capita government financial expenditure, the density of commercial life insurance, the old dependency ratio and health technicians. For convenience, these variables are called HCE, IWGE, INCOME, GFE, DCLI, ODR, CD and HT, respectively. The relevant definitions and explanations of variables are shown in Table 1.

The data on per capita individual HCE, per capita income, per capita government financial expenditure, and the old dependency ratio were from the National Bureau of Statistics of China (NBSC). The data of per capita IWGE were obtained from the China statistical yearbook on the environment (CSYE) and the density of commercial life insurance from the yearbook of China’s insurance (YCI). This study also obtained the data on chronic diseases and health technicians from the China health and family planning statistical yearbook (CHFPSY). There are two things to note: First, it is reasonable to use IWGE represented industrial air pollution since IWGE is the primary indicator of industrial air pollution listed in CSYE. As part of IWGE, the sulfur dioxide emission and other indicators will be studied deeply in the future. Second, data on chronic diseases are directly derived from CHFPSY, where the prevalence rates of chronic diseases are a hybrid concept. It is not a specific chronic disease but refers to those who have one chronic disease at least. In addition, the prevalence rates of chronic diseases were based on 2013 data due to the prevalence rate remaining relatively stable, which were 538.8‰ in 2003, and 539.9‰ in 2013 over the age of 65.

The relevant price data were normalized to be in the 2004 constant price. Meanwhile, in order to reduce the dimensional effects and normalizing requirements, all the data are transformed into their natural logarithmic values.

### 2.3. Statistical Characteristics Analysis

The descriptive statistics of all variables are shown in Table 2.

Table 2 shows the summary characteristics of all variables in high-, middle- and low-income regions. The mean of HCE keeps increasing (6.68 in high-income region, 6.32 in middle-income region, and 6.15 in low-income region), which was synchronized with INCOME and different from the change of IWGE. In addition, it is worth noting that the adjusted skew of HCE had a slight skewness, especially in high-income areas. Therefore, the results obtained by traditional OLS mean regression are feasible but may not be accurate. Thus, it is reasonable for the empirical analysis of BQR to be compared with the OLS mean regression.

## 3. Results

### 3.1. Empirical test

#### 3.1.1. Unit Root Test

To check for stationarity of the changes of HCE and other variables, Table 3 reports the results of the panel unit root tests.

As shown in Table 3, the ADF test provided strong evidence (at the 1% significance level) in support of all the variables that were stationary, and only CD at the 10% significance level. Thus, the results of the panel unit root tests support the argument that there are long-run stable relationships among the variables. In addition, this study found that the fixed effect model was superior to the random effect model through the Hausman test and the F test. Furthermore, the pool test (Row 6 in Table 3) implies that there was obvious individual (regional) heterogeneity. To prove the above, this paper applied the empirical research to the whole country and three different regions respectively, which divided all the samples into the whole country: The high-income region, the middle-income region and the low-income region.

#### 3.1.2. Visual Test of MCMC Convergence

In order to ensure the numerical stability, the data of all the variables was necessary to be standardized and an intercept was automatically included. The number of Markov Chain Monte Carlo (MCMC) iterations are set to 5000, and the post-acceptance check shows whether this is sufficient to find convergence in the MCMC chain. Finally, all the parameters must be passed to the Bayes QR function for empirical research.

**Figure 1:**
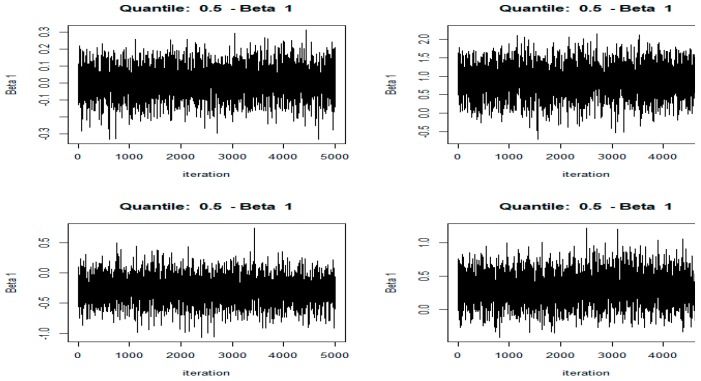
The Markov Chain Monte Carlo (MCMC) chains for the intercept (the upper left), health care expenditure (HCE) (the upper right) and industrial waste gas emission (IWGE) (the lower left), INCOME (the lower right) for the Bayesian quantile regression (BQR). Only the whole country sample as an example were listed here due to space constraints.

Figure 1 was the visual check of MCMC chains of partial variables for BQR. It showed that the MCMC sampler moves rapidly towards a smooth distribution and mixes well, indicating that the MCMC chain has good convergence. In addition, the marginal posterior distributions can be visualized by plotting the histograms of the simulated draws (omitted here). 

### 3.2. Empirical Results of BQR

Table 4 shows the parameter estimation of BQR for the whole quantile series in the whole country, the high-income region, the middle-income region and the low-income region, where some interesting findings were revealed.

Firstly, the income in all sample regions had significant positive impacts on HCE in the whole quantile series. For instance, the influence coefficients were 0.3377 (in the whole country, τ = 0.5), 0.4259 (in the high-income region, τ = 0.5), 0.3165 (in the middle-income region, τ = 0.5), and 0.5759 (in the low-income region, τ = 0.5) respectively. Obviously, income has the largest impact on HCE in the low-income region, which may reveal that HCE was highly sensitive to income for people in the low-income region, but HCE has become an indispensable necessity in the life of people in the high-income region.

Secondly, the variables other than income and IWGE have different effects on HCE. For example, the influence of DCLI on HCE in the high-income region was distinct from the middle- and low- income region. The estimated coefficient of DCLI was −0.1101 (τ = 0.5) in the high-income region but 0.2102 (τ = 0.5) in the low-income region, which may imply that people were more willing to buy health insurance with the function of maintaining and increasing the value instead of HCE because of a greater emphasis on long-term health prevention in the high-income region.

Thirdly, IWGE, the most concerned variable, has very different effects on HCE in the high-, middle-, and low-income regions, which shows double heterogeneity obviously. First, heterogeneity in different regional IWGE has a positive influence on HCE in the high-and middle- income region, while it has a negative influence in the low-income region. For example, the estimated coefficients of IWGE were 0.0028 in the high-income region (τ = 0.5), and 0.0138 in middle-income region (τ = 0.5), which implies that the correlation between environmental pollution and physical health has already attracted attention for all people. On the contrary, the estimated coefficient of IWGE was -0.0981 in low-income region (τ = 0.5), which reflected that people in the low-income region were prone to ignore environmental pollution and inadequate prevention of their own health in the pursuit of economic growth. Second, there is heterogeneity in different quantiles. As shown in Table 4, the influence of IWGE on HCE at high quantiles was significantly lower than low quantiles in the whole country, but the influence of IWGE on HCE at high quantiles was significantly higher than low quantiles in the high-income region.

Finally, it is important to note that, IWGE has little effect on HCE in all the regions compared to the income variable, whether positive or negative, which reflected that people have not paid enough attention to the relationship between environmental pollution and health, although this is slowly changing.

### 3.3. Comparison of Various Empirical Methods

Table 5 shows the regression results (Variable = IWGE) of the different approaches including BQR, BLR, QR and OLS. From the results in Table 5, it was found that there was an obvious difference between the results of BQR and OLS. According to the results of OLS, IWGE had a positive influence on HCE in the whole country but a negative influence in all of the three different regions. This conclusion must be perverse and it may mean that the OLS regression results have some deviations. On the other hand, BQR showed that IWGE had a positive influence on HCE in the high-income region and a negative influence on HCE in the low-income region. This conclusion was basically consistent with the previous articles, such as Yu H, et al. (2018) [18]. Therefore, the results of BQR were more accurate than OLS, which may be due to macro data or the small samples sizes used in this paper.

**Figure 2:**
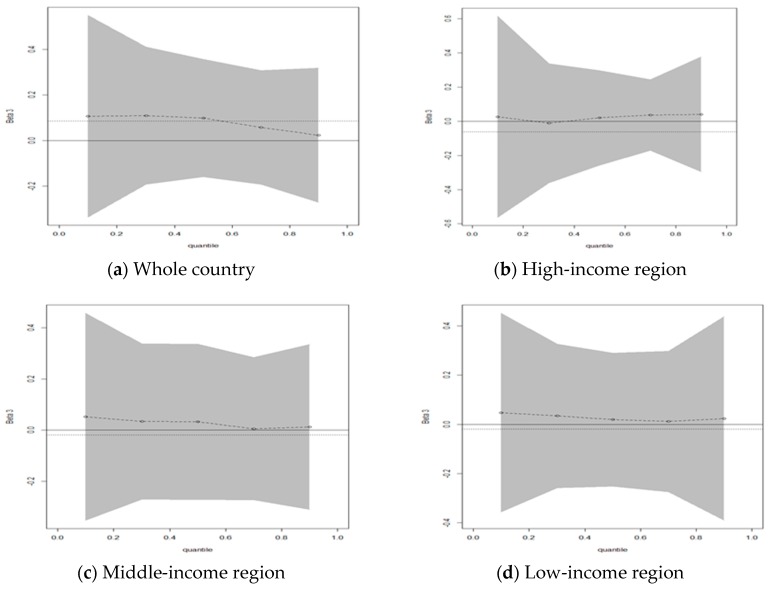
The quantile plots for the Bayes quantile regression and the dotted lines represent the ordinary least squares (OLS) estimate (Variable = IWGE).

In addition, another important feature of BQR could also be found in Figure 2. That is, BQR provided more information for all quantiles over OLS. Figure 2 presents the quantile plots of the variables in the BQR model compared with OLS in the high-, middle-, low-income regions. Here, a full line was added to indicate zero and a dotted line was added to indicate the OLS estimate. The BQR obtained the estimates of all the quantiles and the upper/ the lower values, which is its advantage over OLS. Meanwhile, to save space, not all plots for the four variables are printed here. 

As shown in Figure 2, heterogeneity on the different quantiles was obvious. Furthermore, there was a typical double heterogeneity in the influence of IWGE on HCE. Firstly, significant heterogeneity exists across the whole country. As shown in Figure 2a, the influence of IWGE on HCE at high quantiles was significantly lower than the low quantiles. Secondly, significant heterogeneity exists among the high-, middle-, low-income regions. First, the influence of IWGE on HCE at high quantiles was significantly higher than low quantiles in the high-income region as shown in Figure 2b. Second, the influence of IWGE on HCE at high quantiles was significantly lower than low quantiles in the middle- and low-income regions as shown in Figure 2c and Figure 2d. The relevant conclusions were consistent with the estimated findings in Table 5.

## 4. Discussion

Although the relationship between environmental pollution, especially IWGE and HCE, has attracted great attention from scholars all over the world. There are only a few studies which provide the BQR method to this field, especially using macro data. This paper attempts to introduce the BQR method to the research field of the influence of IWGE on HCE, and revealed some important findings.

Firstly, the double heterogeneity on the different quantiles in the influence of IWGE on HCE was very obvious as shown in Figure 2 and Table 4. On the one hand, the influence of IWGE on HCE at high quantiles was significantly lower than the low quantiles across the whole country. On the other hand, significant heterogeneity exists among the high-, middle-, low-income regions. For instance, the influence of IWGE on HCE at the high quantiles was significantly higher than the low quantiles in high-income regions, but significantly lower than the low quantiles in the middle- and low-income region. Double heterogeneity reveals that people in the high-, middle-, low-income regions have significantly different understandings of environmental and health problems. In particular, people of low-income regions were prone to ignore the environmental pollution and the inadequate prevention of their own health in the pursuit of economic growth. Furthermore, IWGE has little effect on HCE in all the regions compared to the income variable, whether positive or negative, which reflected that people have not paid enough attention to the relationship between environmental pollution and health, although this is slowly changing. Therefore, the government should strengthen media and publicity works on the relationship between environmental pollution and health to improve civic awareness of environmental protection and health prevention, especially in the low-income region. These conclusions are basically similar to previous studies, such as Yu H, et al. (2018) [20] and Lu, Z, et al. (2017) [26].

Secondly, the BQR method, as a new empirical method used to compare previous literature with Chinese samples, is reasonable and feasible for the study in our research field. BQR shows some of its unique characteristics or advantages over the traditional empirical methods. As shown in Table 5, the results of BQR were more accurate than OLS, especially in macro data or small sample sizes. In addition, BQR provided more information for all quantiles, such as double heterogeneity as shown in Figure 2. However, there is some confusion in comparing BQR with other methods. For example, in Table 5, IWGE positively correlates with HCE through BQR, but negatively correlates through QR in the high-, and middle-income regions (tau = 0.5/mean). It may be because the sample size is not large enough, which may cause the results of the Bayesian analysis to be slightly different from those of traditional methods. It should be noted that the authors do not want to prove the BQR method is very superior to traditional empirical methods, but introduce the BQR method into this research field as a new empirical method on the basis of previous studies to expand the discussion space of this field.

Nonetheless, there is still some room for further discussion and expansion when the BQR method is used in this field. However, it has been investigated intensively theoretically and in many other fields, such as biomedical applications, for example, in the choice of data properties and the sample size, the calculation of the prior distribution and the posterior density function [32]. In addition, this study can take into account more variables such as family, age, gender, etc., in future studies if the data acquisition conditions allow. Furthermore, the authors are expected to do more in-depth research in the future research. For example, the authors intend to also try to use simulation according different scenarios to enrich comparisons between the different methods.

## 5. Conclusions

In summary, our findings may contribute to some useful information on the impact of IWGE on HCE in different regions of China. For example, the double heterogeneity on the influence of IWGE on HCE was obvious, which revealed that people in the high-, middle-, low-income regions have a significantly different understanding of environmental pollution and health prevention. The present study suggested that the government should strengthen to improve the civil awareness of environmental protection and health problems, especially in the low-income region. Meanwhile, it was also verified that the BQR method was applicable in this topic and can provide more information than the traditional empirical method. All these conclusions enrich and expand our discussion space. 

## Figures and Tables

**Table 1:** The definition of the variables.

Variable Types	Variable Name	Variable Definition
Dependent variable	ln*HCE*	Per capita health expenditure in each region (yuan) in the form of natural logarithm
Environment pollution variables	ln*IWGE*	Per capita IWGE in each region (ton/10 thousand people) in the form of logarithm
Economic variables	ln*INCOME*	Per capita income in each region (yuan) in the form of natural logarithm
Public service variables	ln*GFE*	Per capita government financial expenditure in each region (yuan) in the form of natural logarithm
	ln*HT*	Number of health technicians per thousand population in each region in the form of natural logarithm
Social variable	ln*DCLI*	Density of commercial life insurance in each region in the form of natural logarithm
Family and personal variables	ln*ODR*	Old dependency ratio in each region in the form of natural logarithm
	ln*CD*	The number of chronic disease each region (1000 people) in the form of natural logarithm

**Table 2:** Summary statistics (after log processing).

Variables	Mean	SD	Skew	Kurtosis	Mean	SD	Skew	Kurtosis	Mean	SD	Skew	Kurtosis
	High-Income Region				Middle-Income Region				Low-Income Region			
HCE	6.68	0.48	−0.15	−0.64	6.32	0.54	−0.09	−0.82	6.15	0.54	0.01	−0.62
INCOME	9.73	0.51	−0.21	−0.59	9.22	0.45	−0.11	−1.19	9.03	0.48	−0.04	−1.23
IWGE	1.32	0.52	0.21	−0.12	1.03	0.63	0.2	−0.6	1.32	0.7	0.23	−0.55
DCLI	6.76	0.82	0.2	−0.49	6.03	0.58	−0.34	−0.73	5.74	0.71	−0.31	−0.71
GFE	−0.45	0.74	−0.23	−0.88	−0.9	0.71	−0.31	−1.04	−0.7	0.77	−0.13	−0.85
ODR	2.57	0.2	−0.01	−0.84	2.53	0.14	−0.13	−0.6	2.43	0.2	0.42	−0.33
CD	6.82	0.69	0.08	−1.26	6.98	0.64	−1.43	1.5	6.47	0.82	−0.86	−0.42
HT	1.7	0.4	0.32	−0.19	1.42	0.24	−0.3	−0.94	1.41	0.3	−0.39	−0.68

Note: The sample size for the whole country is equal to the sum of the three different regions. HCE, INCOME, IWGE, DCLI, GFE, ODR, CD and HT stand for health care expenditure, income, industrial waste gas emission, the density of commercial life insurance, government financial expenditure, the old dependency ratio, chronic diseases, and health technicians respectively.

**Table 3:** The results of the panel unit root test—Augmented by the Dickey-Fuller test (ADF).

Variable	Dickey-Fuller	Variable	Dickey-Fuller	Variable	Dickey-Fuller
HCE	−8.638 ***	GFE	−9.062 ***	CD	−3.186 *
INCOME	−8.827 ***	ODR	−4.293 ***	HT	−6.409 ***
IWGE	−5.952 ***	DCLI	−7.161 ***	——	——
Hausman test	Chisq:63.365(*p*-value: 0.000) ***		F test	F:35.676(*p*-value: <0.000) ***	
Pooltest (effect = “individual”)	F:2.644(*p*-value: 0.000) ***		Pooltest (effect = “time”)	F:0.7186(*p*-value: 0.9566)	

Note: “*” indicates *p*-value < 0.10, “**” indicates *p* < 0.05, “***” indicates *p* < 0.01.

**Table 4:** The Bayes estimate of different quantiles between different income regions. (τ = quantile).

Region	Variables/Quantile	τ = 0.1	τ = 0.3	τ = 0.5	τ = 0.7	τ = 0.9
Whole country	INCOME	0.3874 **	0.3291 **	0.3377 **	0.3362 **	0.3679 **
	IWGE	0.1041 **	0.1067 **	0.1023 **	0.0638 **	0.0229 **
	DCLI	0.0584 **	0.1219 **	0.1897 **	0.2011 **	0.2461 **
	GFE	0.2341 **	0.2791 **	0.2571 **	0.2795 **	0.2671 **
	ODR	0.0140 **	0.0473 **	0.0562 **	0.0434 **	0.0287 **
	CD	0.1308 **	0.0778 **	0.0297 **	0.0128 **	−0.0294 **
	HT	0.2125 **	0.219 **	0.1902 **	0.184 **	0.157 **
High-income region	INCOME	0.3723 **	0.4865 **	0.4259 **	0.4720 **	0.4544 **
	IWGE	0.0099 **	0.0121 **	0.0028 **	0.0264 **	0.0296 **
	DCLI	−0.1042 **	−0.1771 **	−0.1101 **	−0.0137 **	0.0693 **
	GFE	0.4689 **	0.4285 **	0.4603 **	0.3504	0.3152 **
	ODR	0.0374 **	0.05 **	0.0521 **	0.0525 **	0.0348 **
	CD	0.0561 **	−0.0101 **	−0.0446 **	−0.0879 **	−0.1078 **
	HT	0.1811 **	0.2142 **	0.1997 **	0.1592 **	0.1305 **
Middle-income region	INCOME	0.2403 **	0.1661 **	0.0657 **	0.0879 **	0.3003 **
	IWGE	0.0534 **	0.0492 **	0.0138 **	0.0126 **	0.0323 **
	DCLI	0.2657 **	0.2972 **	0.3165 **	0.3052 **	0.2063 **
	GFE	0.1901 **	0.2123 **	0.3208 **	0.3411 **	0.2169 **
	ODR	0.032 **	0.0177 **	0.0161 **	0.0155 **	0.0178 **
	CD	0.137 **	0.1225 **	0.1259 **	0.0946 **	0.0258 **
	HT	0.2448 **	0.2972 **	0.3088 **	0.2759 **	0.2634 **
Low-income region	INCOME	0.5481 **	0.5904 **	0.5759 **	0.5691 **	0.5023 **
	IWGE	−0.0147 **	−0.0822 **	−0.0981 **	−0.1107 **	−0.0411 **
	DCLI	0.2117 **	0.24231 **	0.2102 **	0.2067 **	0.152 **
	GFE	0.1353 **	0.1005 **	0.1254 **	0.1382 **	0.1557 **
	ODR	−0.0028 **	0.0091 **	0.0095 **	0.0177 **	0.0445 **
	CD	−0.0953 **	-0.164 **	−0.1504 **	−0.1511 **	−0.1144 **
	HT	0.1268 **	0.1303 **	0.1656 **	0.1717 **	0.2123 **

Note: All of the outcomes are 95% credible interval (the 95% confidence interval in the quantile regression had the same meaning as *p* < 0.05 in the conditional mean regression such as OLS, so ** were added in this table.). The number of burn in draws: 1000, Number of retained draws: 4000. INCOME, IWGE, DCLI, GFE, ODR, CD and HT stand for income, industrial waste gas emission, the density of commercial life insurance, government financial expenditure, the old dependency ratio, chronic diseases, and health technicians respectively.

**Table 5:** A comparison of the estimation results of various empirical methods (Variable = IWGE, tau = 0.5/mean).

Region	Model	Estimate	Model	Estimate
Whole country	OLS	0.0854 *** (0.0225)	QR	0.1043 ** (0.0154)
	BLR	0.0855 *** (0.0226)	BQR	0.1023 ** (0.0393)
High-income region	OLS	−0.0610 * (0.0413)	QR	−0.0427 ** (0.0757)
	BLR	−0.0607 *** (0.0416)	BQR	0.0028 ** (0.0661)
Middle-income region	OLS	−0.0191 * (0.0367)	QR	−0.0282 ** (0.0456)
	BLR	−0.0190 *** (0.0371)	BQR	0.0138 ** (0.0014)
Low-income region	OLS	−0.2671 *** (0.0480)	QR	−0.3041 ** (0.0154)
	BLR	−0.266 *** (0.0491)	BQR	−0.0981 ** (0.0093)

Note. “***” indicates statistical significance level at 1%. “**” indicates statistical significance level at 5%. “*” indicates statistical significance level at 10%. Both the outcomes of QR and BQR were 95% credible interval (**), In BQR, Number of burn in draws: 1000, Number of retained draws: 4000. OLS: Ordinary least squares; QR: Quantile regression; BLR: Bayesian linear regression; BQR: Bayesian quantile regression.

## Abbreviations

BQR: Bayesian quantile regression; OLS: Ordinary least squares; QR: Quantile regression; BLR: Bayesian linear regression; HCE: health care expenditure; IWGE: industrial waste gas emission.

## References

[1] Dong F., Zhang S., Long R., Zhang X., Sun Z. (2019). Determinants of haze pollution: An analysis from the perspective of spatiotemporal heterogeneity. J. Clean. Prod..

[2] Dong F., Yu B., Pan Y. (2019). Examining the synergistic effect of CO2 emissions on PM2.5 emissions reduction: Evidence from China. J. Clean. Prod..

[3] Guo Z., Liu H., Zhang D., Yang J. (2017). Green Supplier Evaluation and Selection in Apparel Manufacturing Using a Fuzzy Multi-Criteria Decision-Making Approach. Sustainability.

[4] Wang H., Xu J., Liu X., Sheng L., Zhang D., Li L., Wang A. (2018). Study on the pollution status and control measures for the livestock and poultry breeding industry in northeastern China. Environ. Sci. Pollut. Res..

[5] Xu X., Chen L. (2019). Projection of Long-Term Care Costs in China, 2020–2050, Based on the Bayesian Quantile Regression Method. Sustainability.

[6] Wang H., Xu J., Sheng L., Liu X. (2018). Effect of addition of biogas slurry for anaerobic fermentation of deer manure on biogas production. Energy.

[7] Lu X., Yao T., Fung J.C., Lin C. (2016). Estimation of health and economic costs of air pollution over the Pearl River Delta region in China. Sci. Total Environ..

[8] Xu X., Chen L. (2019). Influencing factors of disability among the elderly in China, 2003–2016: Application of Bayesian quantile regression. J. Med. Econ..

[9] Spix C., Wichmann H.E. (1996). Daily mortality and air pollutants: Findings from Koln Germany. J. Epidemiol. Commun. Health.

[10] Xie W., Li G., Zhao D., Xie X., Wei Z., Wang W., Wang M., Li G., Liu W., Sun J. (2015). Relationship between fine particulate air pollution and ischaemic heart disease morbidity and mortality. Heart.

[11] Mazidi M., Speakman J. (2017). Ambient particulate air pollution (PM2.5) is associated with the ratio of type 2 diabetes to obesity. Sci. Rep..

[12] Nayak T., Chowdhury I.R. (2018). Health damages from air pollution: Evidence from open cast coal mining region of Odisha, India. Ecol. Economy Soc..

[13] Li L., Lei Y., Pan D., Yu C., Si C. (2016). Economic evaluation of the air pollution effect on public health in China’s 74 cities. SpringerPlus.

[14] Liu K., Shang Q., Wan C. (2018). Sources and health risks of heavy metals in PM2.5 in a campus in a typical suburb area of Taiyuan, North China. Atmosphere.

[15] Ridker R. (1967). Economic Costs of Air Pollution: Studies in Measurement.

[16] Wordly J., Walters S., Ayres J.G. (1997). Short term variations in hospital admissions and mortality and particulate air pollution. Occup. Environ. Med..

[17] Mead R.W., Brajer V. (2005). Protecting China’s children: Valuing the health impacts of reduced air pollution in Chinese cities. Environ. Dev. Econ..

[18] Narayan P.K., Narayan S. (2008). Does environmental quality influence health expenditures? Empirical evidence from a panel of selected OECD countries. Ecol. Econ..

[19] Remoundou K., Koundouri P. (2009). Environmental effects on public health: An economic perspective. Int. J. Environ. Res. Public Health.

[20] Hao Y., Liu S., Lu Z., Huang J., Zhao M. (2018). The impact of environmental pollution on public health expenditure: Dynamic panel analysis based on Chinese provincial data. Environ. Sci. Pollut. Res..

[21] Sami C., Kais S. (2017). The dynamic links between carbon dioxide (CO_2_) emissions, health spending and GDP growth: A case study for 51 countries. Environ. Res..

[22] Ghosh S. (2010). Examining carbon emissions economic growth nexus for India: A multivariate cointegration approach. Energy Policy.

[23] Amiri A., Ventelou B. (2012). Granger causality between total expenditure on health and GDP in OECD: Evidence from the Toda Yamamoto approach. Econ. Lett..

[24] Soheila K., Bahman K. (2017). Air pollution, economic growth and health care expenditure. Econ. Res. Ekonomska Istraživanja.

[25] Wang K. (2011). Health care expenditure and economic growth: Quantile panel-type analysis. Econ. Model..

[26] Lu Z., Chen H., Hao Y., Wang J., Song X., Mok T.M. (2017). The dynamic relationship between environmental pollution, economic development and public health: Evidence from China. J. Clean Prod..

[27] Zhang H., Niu Y., Yao Y., Chen R., Zhou X., Kan H. (2018). The Impact of ambient air pollution on daily hospital visits for various respiratory diseases and the relevant medical expenditures in Shanghai, China. Int. J. Environ. Res. Public Health..

[28] Jerrett M., Eyles J., Dufournaud C., Birch S. (2003). Environmental influences on health care expenditures: An exploratory analysis from Ontario, Canada. J. Epidemiol. Commun. Health.

[29] Chaabouni S., Zghidi N., Mbarek M.B. (2016). On the causal dynamics between CO_2_ emissions, health expenditures and economic growth. Sustain. Cities Soc..

[30] Apergis N., Gupta R., Lau C.K.M., Mukherjee Z. (2018). U.S. state-level carbon dioxide emissions: Does it affect health care expenditure?. Renew. Sustain. Energy Rev..

[31] Tian F., Gao J., Yang K. (2016). A quantile regression approach to panel data analysis of health-care expenditure in Organisation for economic cooperation and development countries. Health Econ..

[32] Benoit D.F., den Poel D.V. (2017). bayesQR: A Bayesian approach to quantile regression. J. Stat. Softw..

[33] Koenker R., Basset G. (1978). Regression quantiles. Econometrica.

[34] Barrodale I., Roberts F.D.K. (1973). An improved algorithm for discrete L1 linear approximations. SIAM J. Numer. Anal..

[35] Koenker R., Machado J.A.F. (1999). Goodness of fit and related inference processes for quantile regression. J. Am. Stat. Assoc..

[36] Yu K., Moyeed R.A. (2001). Bayesian quantile regression. Stat. Probab. Lett..

[37] Yu K., Zhang J. (2005). A three-parameter asymmetric Laplace distribution and its extension. Commun. Stat. Theory Methods..

[38] Omri A. (2013). CO_2_ emissions, energy consumption and economic growth nexus in MENA countries: Evidence from simultaneous equations models. Energy Econ..

[39] Hansen A., Selte H. (2000). Air pollution and sick-leaves: A case study using air pollution data from Oslo. Environ. Res. Econ..

[40] Sriram K., Ramamoorthi R.V., Ghosh P. (2013). Posterior consistency of Bayesian quantile regression based on the Misspecified asymmetric Laplace density. Bayesian Anal..

[41] Yang Y.W., Wang H.J., He X.M. (2016). Posterior inference in Bayesian quantile regression with asymmetric Laplace likelihood. Int. Stat. Rev..

